# *‘We are all scared for the baby’*: promoting access to dental services for refugee background women during pregnancy

**DOI:** 10.1186/s12884-015-0787-6

**Published:** 2016-01-21

**Authors:** Elisha Riggs, Jane Yelland, Ramini Shankumar, Nicky Kilpatrick

**Affiliations:** Healthy Mothers Healthy Families Research Group, Murdoch Childrens Research Institute, 50 Flemington Road, Parkville, VIC 3052 Australia; General Practice and Primary Health Care Academic Centre, University of Melbourne, Parkville, VIC 3052 Australia; Monash Health Dental Services, 122 Thomas Street, Dandenong, VIC 3175 Australia; Vascular Biology, Murdoch Childrens Research Institute, 50 Flemington Road, Parkville, VIC 3052 Australia; Department of Paediatrics, University of Melbourne, Parkville, VIC 3052 Australia

**Keywords:** Maternal oral health, Dental care, Pregnancy, Refugees, Focus groups

## Abstract

**Background:**

Vulnerable populations such as people with refugee backgrounds are at increased risk of poor oral health. Given that maternal characteristics play a significant role in the development of dental caries in children, antenatal care offers an opportunity to both provide information to women about the importance of maternal oral health and accessing dental care. Although pregnant women are recognised for ‘priority’ care under Victorian state-government policy, rarely do they attend. This study aims to describe Afghan and Sri Lankan women’s knowledge and beliefs surrounding maternal oral health, barriers to accessing dental care during pregnancy, and to present the perspectives of maternity and dental service providers in relation to dental care for pregnant women.

**Methods:**

One agency comprising both dental and maternity services formed the setting for the study. Using participatory methods that included working with bicultural community workers, focus groups were conducted with Afghan and Sri Lankan refugee background participants. Focus groups were also completed with midwives and dental service staff. Thematic analysis was applied to analyse the qualitative data.

**Results:**

Four community focus groups were conducted with a total of 14 Afghan women, eight Sri Lankan women, and three Sri Lankan men. Focus groups were also conducted with 19 dental staff including clinicians and administrative staff, and with ten midwives. Four main themes were identified: perceptions of dental care during pregnancy, navigating dental services, maternal oral health literacy and potential solutions. Key findings included women and men’s perception that dental treatment is unsafe during pregnancy, the lack of awareness amongst both the midwives and community members of the potential impact of poor maternal oral health and the overall lack of awareness and understanding of the ‘priority of access’ policy that entitles pregnant women to receive dental care cost-free.

**Conclusion:**

This study highlights a significant policy-to-practice gap which if not addressed has the potential to widen oral health inequalities across the life-course. Stakeholders were keen to collaborate and support action to improve the oral health of mothers and their infants with the over-riding priority being to develop inter-service relationships to promote seamless access to oral health care.

**Electronic supplementary material:**

The online version of this article (doi:10.1186/s12884-015-0787-6) contains supplementary material, which is available to authorized users.

## Background

Dental decay in children is a common and costly childhood disease [1, 2]. If left untreated dental caries can cause pain and infection, and can lead to hospitalisation; yet it is preventable. While dental caries are undoubtedly multi-factorial maternal characteristics play a significant role [3, 4]. This may be directly through transfer of cariogenic bacteria, indirectly through family dietary patterns and psychosocial environment or poor maternal health during pregnancy (such as smoking or vitamin D deficiency) can compromise tooth development rendering the child’s teeth more vulnerable to decay [5–8]. Furthermore, poor maternal oral health has been shown to not only increase the likelihood of dental caries in children [9] but may also contribute to poor birth outcomes such as low birth weight and premature births [10].

Improving the oral health of mothers during pregnancy, may benefit their children in two general ways. Firstly, interventions that increase women’s knowledge in relation to their own oral health, such as limiting carbohydrate intake, improving oral hygiene habits and receiving dental care, are likely to impact on how they care for their infant’s oral health [11, 12]. Secondly, an improvement in maternal oral health may reduce the number of *S. mutans* (decay promoting bacteria) present in their mouth thereby reducing the potential risk of transmission of these bacteria to their children [13, 14].

There is the opportunity through the provision of the Australian universal public maternity system, to raise awareness amongst women of the value of optimising their oral health and accessing dental care during pregnancy. Non-dental healthcare providers (i.e. midwives) play a key role in the care for pregnant women and are well placed to facilitate oral health promoting activities in this population. Internationally, evidence-based guidelines support the integration of oral health education and dental referral mechanisms in to the care pathways for pregnant women [15]. Such recommendations suggest that all women should have a comprehensive dental screening and risk assessment during pregnancy. This is supported by Australia’s Clinical Practice Guidelines for Antenatal Care which reports good oral health in pregnancy promotes women’s health, that dental treatment can be safely provided in pregnancy, and outlines the advice antenatal care providers should deliver to women about their oral health during pregnancy [16]. The evidence-based guidelines recommend that all women are advised early in pregnancy to have a dental check-up and treatment if required [16].

Globally there are currently 59.5 million individuals who have been forcibly displaced as a result of persecution, conflict, generalised violence, or human rights violations [17]. Australia is a culturally diverse nation with just over one quarter (26 %) of the population born overseas [18]. Australia currently accepts approximately 13,750 people under the Humanitarian Program per year with around 4,000 allocated to the state of Victoria [19]. Recently arrived refugees to Australia come from many countries including Afghanistan, Iraq, Sri Lanka, Burma and South Sudan.

Refugee populations often have higher risks of a range of physical, psychological and social health problems related to experiences of trauma and stressors associated with settlement and persistent disadvantage in the countries in which they live [20, 21]. Prompt access to dental care can play an important role in the recovery from the refugee experience, in particular for people who may have experienced torture resulting in trauma to the teeth, gums or mouth [22]. In addition, current evidence suggests that this population experience significantly higher levels of oral disease than the general population [23]. These experiences can accumulate over time which may result in unmet oral health needs. Knowing this, refugees may find dental treatment highly stressful because of its invasive nature and associations with past traumatic experience [22]. Previous studies have uncovered some of the barriers for accessing dental care in Australia [24, 25] and more recently engagement with maternity care [26]. These experiences can accumulate over time meaning that people with refugee backgrounds may continue to have unmet and often complex oral health needs long after arrival in their new country. As well as language and cultural challenges, people with refugee backgrounds often have a limited awareness of preventative care and present to dental services with acute dental problems requiring emergency treatment. Previous research has shown that despite mothers’ knowledge of the major causes of poor oral health (diet and oral hygiene) there remains much confusion about child oral hygiene practices and limited oral health literacy which influence child oral health outcomes [27]. Furthermore families of refugee background typically find it challenging to access dental services due to language and transport difficulties as well as perceived costs, wait lists and concerns about the quality of care provided [27]. In addition to poor oral health outcomes, people with refugee backgrounds also experience poor perinatal outcomes [28, 29], the reasons for which are also multifactorial and complex [26].

### The Victorian context

Victorian government policy outlines that pregnant women, children, and refugees and asylum seekers are some of the named ‘priority groups’ for receiving free public dental care [30]. This policy entitles people with a Health Care Card (concession provided by the Australian Government to help with the cost of prescription medicines and government funded services) to be prioritised for the next available appointment for dental care with their course of care provided cost-free. ‘Priority group’ individuals are not placed on the general public dental care wait lists. For pregnant women, their course of care can begin during pregnancy and be completed post birth. Not all refugees and asylum seekers hold Health Care Cards but are still eligible for ‘priority’ dental care.

On arrival to Australia, sub-contracted Humanitarian Settlement Services provide intensive settlement support to people who hold permanent humanitarian visas through a coordinated case management approach for 6-12 months. Case managers support clients to connect with health services, including GPs and refugee health nurses for comprehensive health assessments soon after arrival in Australia, of which oral health is included. Community guides also provide new arrivals with settlement support in their own language during the first six months of resettlement in Victoria, which includes assisting the client to orient themselves to local services. This service is not available for all those on a subclass of a humanitarian visa [31]. Asylum seekers who are living in the community on temporary visas or are in community detention also receive case management support through subcontracted agencies. Different types and levels of support are provided, depending on the individual circumstances of the person.

A high proportion of refugees to Victoria settle in the south-east region of Melbourne. Given the cultural diversity of this region, populations who have more recently settled in the region’s municipalities and with the limitations of project funding, the project reference group chose to include two refugee communities in the study – people from Sri Lanka and Afghanistan. These population groups have been selected for this study for the following reasons (1) local dental service data suggests that there is limited utilisation of these services by pregnant women from these backgrounds while, children from these backgrounds present to the service in early childhood with a need for dental treatment (2) consultation with local service providers located in south east of Melbourne working with refugee families reported high numbers of pregnancies in these cultural groups.

The aim of this study was to investigate understandings of maternal oral health, dental priority groups and information provision from the perspectives of the refugee and asylum seeker community, and dental and maternity care providers in Melbourne, Australia. The objectives of this paper are to (1) describe Afghan and Sri Lankan women’s knowledge and beliefs surrounding maternal oral health and barriers to accessing dental care during pregnancy (2) describe the perspectives of maternity and dental staff in relation to priority dental care for pregnant women of refugee background, and (3) consider the implications of the findings for translation into policy and practice.

## Methods

The Murdoch Childrens Research Institute in partnership with Monash Health Dental Services conducted this study in 2014. A collaborative participatory approach was adopted to explore the perceptions of refugee and migrant women and healthcare providers (e.g. midwives, dental professionals) towards oral health care during pregnancy and their views regarding provision of oral health information and referral pathways to local dental services.

Ethical approval was obtained from the Human Research and Ethics Committees of Monash Health (14011X) and the Royal Children’s Hospital (34010A).

### Setting

The outer south-east region of Melbourne is characterised by population growth, cultural diversity and social disadvantage. Significant numbers of refugee background people from Afghanistan, Sri Lanka, Iran, Sudan and Burma have settled in the region. Most come from non-English speaking countries, live on very low incomes and experience multiple health and social issues.

Monash Health is a large network of primary, secondary and tertiary health care services in the region. ‘Monash Women’s’ health service provides antenatal, intrapartum and postnatal care to approximately 8000 women who give birth annually across three hospitals. All women booked to give birth at one of the Monash Health hospitals see a midwife for at least one pregnancy visit. Women can attend hospital clinics for all their pregnancy care with a midwife and/or medical practitioner; or attend shared maternity care where antenatal care is provided by a general practitioner (GP) with some pregnancy check-ups provided by hospital staff. Around 10 % of all births are to women of refugee background. The ‘Monash Health Dental Service’ operates from 30 community based dental clinics and provides public dental care to 32,000 patients annually.

One of the drivers behind this study was data indicating that the dental service has very small numbers (approximately 1 %) of pregnant women attending despite the large number of births in the region.

### Recruitment and data collection

A project reference group was established to advise the research team (ER, JY, RS, NK) and met three times over the course of the project. Members included maternity and dental service managers, and refugee and early childhood health specialists.

#### Health care provider sample and recruitment

Dental and antenatal care providers from Monash Health were invited to participate in the study. All dental staff from two dental service sites with high numbers of refugee background clients were invited to participate. Similarly, two teams of midwives from two different hospitals providing antenatal care to clients of refugee background were invited to participate. An email invitation to attend a 1 h focus group discussion and plain language statement was sent to all these staff members. The dates, times and location were organised by service managers to ensure clinical obligations were covered. Participation was voluntary and everyone signed a consent form before the focus group started. A semi-structured theme guide was used which included: provision of care to refugee background families including asylum seekers; maternal oral health knowledge; information provision and resources; and inter-service knowledge (Additional file 1). All focus groups were conducted by the primary researcher (ER) with the assistance of a note taker (either NK or JY). Potential ethical issues regarding conducting research in the workplace were considered. No managers were involved in the staff focus groups and it was explained to staff that non-participation would not affect their employment.

#### Community sample and recruitment

A semi-structured theme guide was used which included: access and experience of dental services in pregnancy; maternal oral health knowledge; and access to oral health information (Additional file 1). The questions were pilot tested with bicultural workers prior to the focus groups for cultural sensitivity.

Three bicultural community workers assisted in locating and inviting women who had young children to participate in focus groups. Participant selection criteria included: identifying from Afghanistan or Sri Lanka, living in the south-east Melbourne region and having a child aged 0–3 years. The bicultural workers used both purposeful and snowball strategies to identify, invite and recruit eligible participants through playgroups that they facilitated and their community networks. All participants signed a consent form which was provided in their preferred language of English, Tamil or Dari. Each community worker was paid an honorarium reflecting the hours of time spent working on the project. The community workers and primary researcher (ER) spent considerable time discussing the purpose of the research and issues for consideration including: recruitment, confidentiality, focus group facilitation, interpreting, audio recording, participant reimbursement, catering, location, child care and dissemination of findings.

#### Data analysis

As an introductory activity each community focus group began with collecting demographic data from participants including country of origin, year of arrival in Australia, number and age of children. This information was not audio-recorded but noted in written form. For care providers, data included profession, position, duration of employment with the organisation and site/s located.

All focus groups were audio recorded and transcribed verbatim by an external party. All focus groups were conducted in English and interpreted by the bicultural worker to ensure an English transcript for analysis. Additional field notes were also documented. All transcripts were imported into NVivo 10 for data management [32]. Thematic analysis was utilised and all co-authors read the transcripts and the primary researcher (ER) undertook all transcript coding and categorising [33]. Open coding was used to break down responses to distinct units of meaning; codes were then organised to develop categories providing distinct key themes.

## Results

**Fig. 1:**
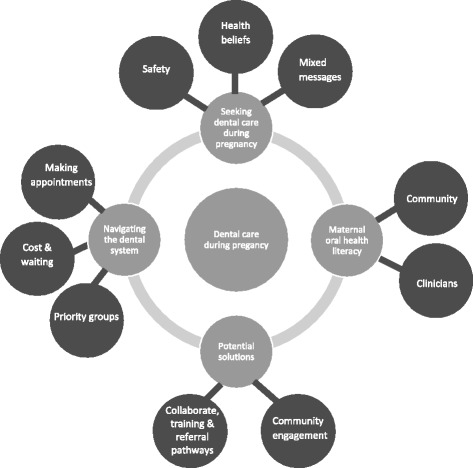
Thematic map of qualitative results

A total of four community focus groups were held. The two Afghan groups had six and eight women in each. The first Sri Lankan group had eight women and the second had two women and three men. The men were husbands or brothers of the women and were interested in learning about services available to their families and were encouraged by the bicultural worker to attend. The Afghan women identified as Hazara and all spoke Dari, had lived in Australia for an average of 7 years (range 2–13 years) and had a total of 47 children between them. All Sri Lankan participants identified as Tamil and were seeking asylum in Australia. They had been in Australia for an average of 1 year (range 6 mths-4 ys, though 7 had been in Australia less than a year) with a total of 23 children between them. Two focus groups were conducted with a total of 19 dental staff including clinicians (dentists, oral health therapists and dental assistants) and administrative staff. Two focus groups were completed with a total of ten midwives. Overall four themes were identified and these are reported here. Figure 1 summarises the identified themes.

### Seeking dental care during pregnancy

Rarely had women visited a local dental service for themselves for prevention when pregnant or at any other time. Mothers were far more likely to have sought out their local dental service when their child was in need of emergency care or treatment. A combination of women’s health beliefs, a fear of dental treatment, and mixed messages from health care providers were significant barriers for seeking dental care during pregnancy.

#### Health beliefs

Across both cultural groups the use of dental services for preventive health reasons was not common.

*Culturally, if we are not in pain we’re not going to access, go to dentist. (Afghan woman)*

This was despite many women reporting they had been, or currently were experiencing dental pain, toothache, bleeding gums and sensitive teeth.

*Because she has got toothaches but her turn has come late (on the waiting list), she crying from the sharp pain. She was told to wait until her turn has come up…go home and come another day. Still she sit at her house and now she’s suffering from that pain. (Afghan woman)*

#### Safety for the unborn child

Overall, there was much confusion surrounding dental attendance during pregnancy. The prevailing message being that it is not safe to have dental treatment during pregnancy for fear of harm to the unborn child.

*I was screaming (with pain), I was walking up and down but I didn’t go to the dentist because I was told that if you have an injection or take medicine or anything it would be bad for your child. We’re all scared in case it has any side effect for the baby. (Afghan woman)*

*Because we think the anaesthetic they give is not good for baby. (Afghan woman)*

In contrast, reassurance by a dental care provider alleviated one woman’s safety concerns as required multiple x-rays to be conducted during her pregnancy.

*When I went for treatment for my teeth I was really concerned about when they took the x-ray because all the time, for six times when I went there, they take x-ray, I was concerned to not affect my baby but the dentist told me we take the x-ray over the throat, it does not affect the baby. (Afghan woman)*

#### Mixed messages

A few women reported inquiring about dental care during pregnancy, yet their GP advised them not to go to the dentist during pregnancy.

*I needed to get a filling done but it was in the third trimester so they asked me to wait and they said “only they can do it after six months after having the baby”.*

***Do you remember which dental service told you that?****(Focus group facilitator)*

*The family doctor, the GP told me that. (Sri Lankan woman)*

This was reaffirmed for a few women who reported dental pain during their pregnancy and sought dental care but were refused appointments, they were unsure why this was the case. Other women also reported going to the dentist when they were pregnant but were not allowed to have injections, fillings and extractions, without receiving an explanation why.

*Yeah, when I was pregnant they wouldn’t see me. When I asked them they said “no, you can’t”. (Afghan woman)*

However, the dental providers reported that pregnant women rarely attend their service for a check-up but stated they welcomed women at any stage of pregnancy. The pregnant women who were seen at the dental service were typically identified when they arrived for emergency treatment for themselves or their child or were identified when they accompanied their child for their usual dental appointment.

*They’ve come in as an emergency and we’ve said “you’re actually a priority” [because you are pregnant] and then we book them in for an exam after that. (Dental provider)*

### Navigating the dental service system

#### Cost and waiting

Regardless of being pregnant, the cost of dental care was commonly reported by Afghan women, to be a barrier to seeking dental appointments. Even with a Health Care Card, the concession co-payment rate of AUS$26.50 was a financial concern for women. Regardless of being pregnant, women were not aware that they could access dental care as a refugee or an asylum seeker as part of the ‘priority’ scheme which meant they would be exempt of the co-payment. There was also confusion about the breadth of treatment provided for this cost and what treatment would require additional fees. Many women reported that they had experienced increased costs associated with fillings, extractions and more complex care. The women were unclear whether these costs had arisen from private or public dental providers as they could not always recall what type of service they had attended. Cost was a significant factor for the women and men who did not have a Health Care Card and were unaware of their eligibility for ‘priority access’ as refugees or asylum seekers. Many Afghan women reported seeking dental care overseas because it is affordable.

*Because it’s expensive we’re not going but some of the others, they are going for treatment overseas like in Afghanistan, Pakistan it is cheap. (Afghan woman)*

Several Afghan women reported being placed on a dental waiting list but then had not heard from the dental service since. The longer they waited the more reluctant they were to proactively seek dental assistance and advice.

#### Making and getting to dental appointments

In the absence of any discernible oral health issues, very few women accessed dental services at any time including whilst pregnant. A woman’s ability to make appointments depended on several factors: their own confidence and ability to speak English, having a family member who spoke English, knowing a health professional (such as a community-based health nurse) who telephoned the service on their behalf, having the correct phone number or knowing where the service was located to make a ‘walk-in’ appointment, and lack of understanding of being a ‘priority’ group for receiving co-payment exempt dental care with no waiting list.

Participants used several means to get to their local dental services. Most walked or used public transport. A small number of women drove themselves or had their husbands drive them, though this was mostly for appointments for their children.

A few women required emergency dental treatment during recent pregnancies. In these instances, women were directed to attend the dental hospital,^1^ located in the city 30 km from their local community-based service. For those women to attend this hospital their husbands were required to take a day off from work to drive them there. For one woman, this occurred six times during her pregnancy.

*During my pregnancy…when I ring [the local dental service] I explained to them I’m in pain and they say “we haven’t got facility for pregnant women, you should go to Royal Dental Hospital”. (Afghan woman)*

Many participants reported receiving letters from dental services in English and being unable to read these. It is unclear if these were letters notifying people of appointments times.

#### Awareness of dental services and priority groups

Whilst all dental staff had good knowledge of the Victorian policy for ‘priority groups’ for dental access, neither the community participants nor maternity staff had any knowledge of this policy. Although the Sri Lankan participants had only been living in the country for a short time, they all should have received a comprehensive health assessment which includes oral health. Despite this provision none had accessed dental care for themselves even though they reported dental problems. A few had accessed dental services for emergency treatment for their children.

Overall none of the midwives were aware that a dental service was located in the same public health service which they worked in.

*No idea. No, we have no information. (Midwife)*

*There isn’t one really [dental service], we have nothing, nowhere to send people. (Midwife)*

Once the policy was explained, the midwives spoke about having seen many pregnant women with poor oral health, who would meet the eligibility criteria. None of the midwives reported ever having referred a woman to a dental service.

*No, we don’t refer. We don’t tell them to do something about going to the dentist. (Midwife)*

*If a woman came in to clinic with strong toothache, I’d say “go see the dentist”. You know, I wouldn’t refer to our dental services. I’d just say “go to your own dentist”. (Midwife)*

Dental providers reported receiving referrals from many external agencies to see several priority groups, but rarely received referrals for pregnant women.

*It [referral letter] clearly states that they’re a refugee or asylum seeker but we don’t see referrals saying “can you see this patient because she is pregnant”, we don’t get referrals like that. (Dental provider)*

Midwives were shocked to realise that they were not aware that a dental service was available within the same organisation in which they were employed. This was despite making referrals to other departments within the organisation (e.g. social work, nutrition and physiotherapy) when women’s needs were identified during pregnancy. These referrals occurred because the services were often co-located which provided opportunities to meet face-to-face which then facilitated relationships to be established with a range of allied health clinicians.

*I think that also goes back to how we refer to physios because we see them on the wards, we see them and we see the social workers because we know them. We know who they are. We have a wonderful working relationship with our social worker and also the physio because we see them often on the ward. We talk to them directly with the concerns we have and we have meetings as well but the dental service is quite a separate identity. (Midwife)*

### Maternal oral health literacy

Although dental care providers were aware of the relationship between maternal oral health, birth outcomes and child oral health, neither the maternity staff nor community participants were. One midwife articulated the relationship between maternal oral health and the potential transmission of cariogenic bacteria leading to poor child oral health.

*Kissing, dummies, food, anything…and then you end up with the bacteria in the baby’s mouth which leads to dental caries. Mind blowing information it is. I only learnt that recently. Amazing.*

***Where did you learn about it?****(Focus group facilitator)*

*I don’t know where I picked it up from, but it was scary. (Midwife)*

Midwives who provided care at different time-points such as antenatal or postnatally noted that despite discussing several preventative health issues with women, at no time during pregnancy or post-birth was maternal oral health discussed.

*Even postnatally when we send women home, we might talk about contraception, talk about the next pregnancy, PAP smears…we don’t have anything about dental, considering how important it is. (Midwife)*

There was a marked lack of knowledge by all community members surrounding maternal oral health and its potential implications, with the exception of one Sri Lankan women who had been told about this by her midwife in her home country. Typically women recalled knowing about illnesses such as the common cold being passed from mother to child but were unaware of oral health issues.

*We only have heard that some infection from mum passed to the baby but not toothache like if you have got a cold or some other problem it’s passed to the baby but never have we heard about this problem affecting the baby. (Sri Lankan woman)*

Most Afghan women could recall information about the causes of poor child oral health, reporting that they received this from their local child health nurse. However, no one had informed them of the importance of their own oral health and its potential implications.

*When we go to the maternal and child health nurse we are just talking about kids not about mum. (Afghan woman)*

Although many of the newly arrived Sri Lankans spoke about their children having poor oral health they were not aware of the causes. Several women and men noted feeding chocolate and lollies to their children since they were babies.

Most community participants reported that if they wanted specific health information that they would ask their GP or, in the case of the Sri Lankan participants, their settlement agency case manager. Everyone - community members and clinicians - requested more information on maternal and child oral health, priority group eligibility and where and how to access dental services.

### Potential solutions

#### Collaboration, communication and referral pathways

All participants suggested that improved communication between maternity and dental health services would promote maternal oral health and improve access to dental services during pregnancy. Both maternity and dental service providers were enthusiastic about working together to find solutions whilst recognising each other’s time constraints and capacity. As a first step clinicians supported the benefits of meeting each other in person, as a way of improving inter-disciplinary communication and developing referral pathways.

*Because they [maternity services] can work with us and we can give them information brochures and I think it would definitely improve the situation. (Dental provider)*

*We are under one umbrella [Monash Health] so we should be able to organise referral pathways. (Dental provider)*

The need for staff training and supporting resources to identify and refer those eligible for priority dental care during pregnancy was universally acknowledged. Midwives were keen to participate in professional development to learn about maternal oral health and the available dental services. Similarly, all dental staff (clinical and administrative) were keen to participate in joint training to ensure that key oral health messages were transparent and consistent across the services. A combination of face-to-face and online training options were supported as midwives often work night shift therefore options for them needed to be considered.

Given the time pressures that exist for midwives working in antenatal care, there was general acknowledgement that they could not be expected to provide extensive oral health advice or undertake oral examinations for all pregnant women. However, being aware of maternal oral health and the importance of dental checks in pregnancy would help them to initiate conversations with women and be confident in providing advice when necessary. Likewise, being knowledgeable of the dental service, priority access policy, and referral pathways would help them to facilitate women’s access to dental care.

*Our only gripe is about the length of time that you have for a booking appointment [the first antenatal appointment]… but certainly, for the midwives to be educated in oral hygiene and what the possibilities are, that’s something that would help. (Midwife)*

Health care providers reported that visual aids such as pictures and videos would be helpful for engaging the community and sharing information about oral health. In addition, dental providers found it valuable to sit with patients in a comfortable setting (i.e. not in the dental chair), accompanied with an interpreter to discuss oral hygiene and post-procedure care.

#### Community engagement

Afghan and Sri Lankan participants suggested coming together in cultural community groups to meet with a credible dental service provider as a means of accessing information in a setting where they are able to ask questions. General practitioners and midwives were reported as a key health care providers during pregnancy who could also provide oral health information and explain how to access dental services. However, community members noted that there was not a lot of time available at these pregnancy appointments for discussion. Community-based maternal and child health nurses were seen as a plausible option but they were perceived as a resource for child health more so than maternal health.

*The GP and the maternal child nurse, if you not ask them they not giving you information because of limit of time. It’s hard for them to tell us. We prefer something like this [discussion group] with a dentist, would be helpful. (Afghan woman)*

Translated written information was also suggested though it was recognised that not everyone could read in their own language but most knew someone who could translate for them.

## Discussion

Poor maternal oral health is a modifiable health condition with adverse implications for perinatal and early childhood oral health. This study has identified barriers that exist for refugee background women to access dental services during pregnancy and the obstacles facing care providers in facilitating access to such services. For women, there is an overwhelming concern for the safety of their unborn child which inhibits them seeking dental care when pregnant, despite this being a key time to receive dental care.

This study is the first to explore the perceptions and experiences of refugee background women about their oral health in pregnancy. The gap in knowledge surrounding oral health during pregnancy has been reported previously in non-immigrant pregnant women [34], immigrant women [35], and prenatal care providers [36]. What remains unclear is whether length of time in Australia and other acculturative factors influence health promotion and prevention knowledge. In this study, the Afghan women who had lived in Australia on average much longer than the Sri Lankan women and men, remained unaware of maternal oral health issues.

Despite the lack of dental attendance by the women in this study, pregnancy remains a timely opportunity to raise the importance of maternal oral health. Whilst evidence for the benefits of periodontal treatment in improving birth outcomes remains inconclusive [10, 37], establishing a dental home for these women during pregnancy is likely to improve the oral health outcomes for both mother and future child/ren. This would promote the prevention of ECC. Furthermore, access to dental services for refugee background families is reported to assist with settlement by alleviating health issues and providing opportunities for enhancing oral health literacy by providing information and advice [22].

The results of this study demonstrate that accessing preventative dental care is a new concept for people of refugee background, even though the need for treatment is high. This attitude to preventive dental care has been reported previously in these communities [24]. Both this study and earlier work, suggest that messages surrounding the importance of child oral health do appear to be reaching the community, however awareness of maternal oral health remains very limited. Whilst several barriers to accessing care for immigrant mothers have been identified [24], antenatal care providers are well placed to ameliorate these challenges and facilitate access to oral health services. Strategic engagement requires comprehensive understanding of community beliefs and perspectives as there is evidence that women perceive poor oral health during pregnancy as normal [38].

Despite the existence of national and state guidelines and policy [16, 30] the midwives in this study were unaware of these. Developing partnerships and strengthening relationships between the services would build capacity for interdisciplinary communication. In addition it would facilitate referrals and provide opportunities for discussion between dental and antenatal clinicians should dental treatment be required during pregnancy. The need for midwifery education and dental service referral pathways have previous been identified [36].

There were mixed message surrounding the safety of dental treatment during pregnancy with some women having been told to delay their dental treatment until after birth by their GP. In contrast a recent study has shown that obstetricians are knowledgeable of the implications of periodontal disease and adverse pregnancy outcomes and support treatment during pregnancy, although they themselves did not feel skilled to recognise oral disease in their patients [39]. It is common for non-dental professionals to be wary of providing oral health advice and/or conducting oral examinations, believing that it is the role of the dental profession [40]. Although, neither general practitioners nor obstetric clinicians were consulted in this study, they do provide a high proportion of antenatal care. Engagement with and training for these service providers should also be considered.

Refugees are considered a ‘hard to reach’ population for both health services and research studies. For example, Thomas et al. investigated oral health practices in pregnancy but did not use research methods that are inclusive of non-English speaking participants [41]. Economic and other skilled migrants, whilst also facing challenges settling in their new country don’t often experience similar vulnerabilities associated with torture and trauma that people with refugee backgrounds often do. A strength of this study was the involvement of both the community (refugee and asylum seeker women and men) and the dental and midwifery sectors. In addition, being guided by the bicultural workers on all aspects of the study ensured the methodology for community engagement was culturally competent [42]. However, there are limitations. Limited funding and time constraints, meant that our consultations only took place across two geographic sites with two communities and involved only one health service; this may have impacted on the breadth of the consultative process.

## Conclusion

Despite national guidelines and priority of care entitlements for dental care, maternal oral health is a neglected aspect of pregnancy care. Whilst non-immigrant pregnant women face barriers accessing dental care, refugees and asylum seekers face additional barriers in accessing such services. Vulnerable populations such as these are at high risk of developing oral disease and have limited oral health literacy. Utilising participatory methods, this study is the first of its kind to work with Afghan and Sri Lankan refugee background women and both dental and maternity service providers to explore their perspectives of maternal oral health and use of dental services during pregnancy.

This study has identified the gap in both the community and midwives knowledge of the ‘priority access’ policy for public dental services and understanding of maternal oral health. All stakeholders were keen to collaborate and support action to improve the oral health of mothers and their children. The solutions identified by the clinical participants align with international evidence that multidisciplinary approaches are needed to address complex issues of oral health inequality [43]. A multidisciplinary approach to maternal and child oral health is necessary, with priority placed on the development of inter-service relationships to promote seamless access to oral health care. Co-designed approaches to addressing these service gaps could include initiatives that build the capacity of maternity staff to have conversations with women about the importance of oral health and alleviate fears of dental care in pregnancy; particularly those with low health literacy and/or of refugee background; improved communication systems and referral pathways between maternity and dental services; and sustainable platforms to support maternity-dental service initiatives including joint training and professional development. Collaboration and innovation is necessary to address the significant policy-to-practice gap and reduce oral health inequalities across the life-course.

## Additional file

Additional file 1:
**Focus group guides.** (DOCX 16 kb)

## Footnotes

1 The Royal Dental Hospital provides treatment for patients with more complex dental needs and in particular provides treatment under general anaesthesia.
