# Supplementary material for: ‘We are all scared for the baby’: promoting access to dental services for refugee background women during pregnancy
Source: BMC Pregnancy Childbirth. 2016 Jan 21;16:12. doi: 10.1186/s12884-015-0787-6 (PMC4722780; doi:10.1186/s12884-015-0787-6)
Supplement: Additional file 1: — Focus group guides. (DOCX 16 kb) [file 12884_2015_787_MOESM1_ESM.docx]

**Community focus group guide**

*Access to dental services in Victoria*

- Have you ever visited a dentist in Australia? Where was it? Do you know where your closest dentist is?
- How did you hear about the service and that location?
- How did you get there? Did you use public transport, walk, drive, someone drove you in a car?
- Have you had any problems getting a dental appointment or getting to a dental service when you needed to? Is there anything that stopped you from going?
- Have you used a dental service when you were pregnant?
- Would you have dental treatment whilst you were pregnant?
- Have you taken your youngest child to the dentist? Why/why not?

*Maternal and child oral health*

- Can you recall any issues you had with your oral health/mouths/gums/teeth during pregnancy?
- Please tell me what you know about mother’s oral health in pregnancy and the impact it can have on 1) the baby’s health when he/she is born and 2) the child’s own dental health.
- Do you know whether your child’s oral health is affected by your own oral health?

*Oral Health information*

- Do you know about public and private dental services?
- Do you go to the dentist for check-ups or to have treatment?
- Do you know about ‘priority groups’? Explain: Pregnant, children, refugee, asylum seeker etc.
- Have you been provided with any dental information for yourself or your children? If yes who? What was the information provided? Format? Useful?
- Where would you go, or who would you ask, if you wanted dental information for you or your child?
- What do you think is the best way to find out about how to keep your and your children’s teeth healthy? When is the best time to give you dental information?

**Maternity sector focus group guide**

***Access to maternity services***

- We are working with the Sri Lankan and Afghan communities; do you provide care to these people?
- Are patients given a choice of antenatal provider such as midwife/GP/obstetrician whether they are male/female?
- Are people referred to you/your clinic? How does this work?
- Do you make referrals? What for? Who to?
- Do clients see the same care provider each time the come for antenatal care??
- What do you think make patients want to come back to your service?
- How do they hear about the maternity service here? Do you know how they get to the hospital/clinic? Drive, walk, public transport
- What happens when someone comes as an emergency/complications? Interpreter?
- How do patients of non-English speaking background book appointments with your service? Do they receive reminders about their appointments? Do you use telephone interpreters? Do people attend? What happens if they fail to attend? Eg followed up, re-booked

***Knowledge of local Dental Services***

- Do you refer women to dental services? Where? Do you think women attend? If not, what are the reasons why? Do you follow up on the outcome of referrals?
- Can you tell me what you know about ‘priority groups’ for accessing public dental services?
- Do you ever refer women to the dental service? As a priority group?
- Would you know if your patient was a refugee or an asylum seeker?

***Relationship between poor maternal oral health and child oral health outcomes***

- Is maternal oral health covered in antenatal care provision? Please explain.
- Are you aware of any protocol, evidence, clinical guidelines around the relationship between maternal oral health and child oral health outcomes?
- Have you noticed that pregnant women, particularly refugees, have specific needs regarding their dental care? How do you respond to this? How do women respond to you providing dental information?

***Information and resources***

- Do you use translated information with patients? What kind of information? Where do you get this from? Is it useful?
- Do you think your patients of non-English speaking background understand the information and advice you give? What makes you think this? Why/why not?
- Do you require other information or resources to help explain oral health or service issues to people? What do you think would be useful?

***Service strengthening***

- How do you feel about the support you receive for providing dental advice to refugee/asylum seeker women?
- Have you ever completed training on working with refugee/asylum seeker clients? Tell me more
- Some hospitals have started to do targeted training with midwives which includes encouraging them to refer their pregnant mothers to dental services. If this were to happen here, what do you think the impact would be? Would this training interest you?
- What would you need to feel confident in providing oral health advice to pregnant women? What sort of training, practice and resources would be helpful to you?
- Do you know anyone from the dental service? Would it be useful to meet anyone? To learn how their service works?
- Do you think it’s important to create/strengthen relationships between maternity and dental services? Prompts. How could this happen? Who would be important people to involve? Should there be a dental professional working onsite with midwives? Would guidelines/protocols/referral pathways be useful ways of establishing this?
- Do you work with any other agencies? Which ones? What support does this offer? Do you think there are some agencies that would be beneficial to work with?
- Are there any organisational changes that you think might help us to improve the care and outcomes for this group?

**Dental sector focus group guide**

***Access to dental services***

- We are working with the Sri Lankan and Afghan communities; do you provide care to these people?
- Do you find people mostly attend for check-ups, treatment, emergencies?
- How do they hear about your service? Do you know how they get to the clinic?
- Are patients given a choice of provider such as their dentist/therapist and whether they are male/female?
- Are people referred to you? How does this work?
- Do you make referrals? What for? Who to?
- Do clients see the same care provider each time the come to the service? Do you see the same members from one family?
- What happens when someone comes in with an emergency?
- Can you tell me what you know about ‘priority groups’ for dental care?
- Do you see many refugees, asylum seekers pregnant women and children who use the service as priority groups?
- Would you know if your patient was of refugee background or an asylum seeker?
- What do you think make patients want to come back to your service?
- How do patients of non-English speaking background book appointments and receive reminders about their appointments? Do you use telephone interpreters? Do people attend? What happens if they fail to attend? Eg followed up, re-booked

***Relationship between poor maternal oral health and child oral health outcomes***

- We are focussing on working with pregnant women to improve their health and the health of their baby. What’s your experience of working with pregnant women? How do you feel about it?
- Do you think there are benefits of working with the mother/pregnant mother and then the baby/child? Either for the mother and/or their baby/child.
- Are you aware of any protocol, evidence, guidelines around the relationship between maternal oral health and child oral health outcomes? Clinical guidelines for caring for pregnant women?
- Have you noticed that pregnant women, particularly refugees, have specific needs regarding their dental care? How do you respond to this?

***Information and resources***

- Do you use translated information with patients? What kind of information? Where do you get this from? Is it useful?
- Do you think your patients of non-English speaking background understand the information and advice you give? What makes you think this? Why/why not?
- Do you require other information or resources to help explain oral health or service issues to people? What do you think would be useful?

***Service strengthening***

- How do you feel about the support you receive for providing dental care to refugee/asylum seeker/pregnant women/young children?
- Most of you have completed the Targeted Education Program on refugee oral health, was this helpful? In what way?
- Some hospitals have started to do targeted training with midwives which includes encouraging them to refer their pregnant mothers to dental services. If this were to happen here, what do you think the impact would be?
- Do you currently work with any other agencies? Which ones? What support does this offer? Do you think there are some agencies that would be beneficial to work with?
- Are there any organisational changes that you think might help us to improve the care and outcomes for this group?
